# Reporting and handling of missing data in predictive research for prevalent undiagnosed type 2 diabetes mellitus: a systematic review

**DOI:** 10.1186/s13167-015-0028-0

**Published:** 2015-03-11

**Authors:** Katya L Masconi, Tandi E Matsha, Justin B Echouffo-Tcheugui, Rajiv T Erasmus, Andre P Kengne

**Affiliations:** Division of Chemical Pathology, Faculty of Health Sciences, National Health Laboratory Service (NHLS) and University of Stellenbosch, Cape Town, South Africa; Non-Communicable Diseases Research Unit, South African Medical Research Council, PO Box 19070, , Tygerberg, 7505 Cape Town, South Africa; Department of Biomedical Technology, Faculty of Health and Wellness Sciences, Cape Peninsula University of Technology, Cape Town, South Africa; Hubert Department of Public Health, Rollins School of Public Health, Emory University, Atlanta, GA USA; Department of Medicine, MedStar Health System, Baltimore, MD USA; Department of Medicine, University of Cape Town, Cape Town, South Africa

**Keywords:** Predictive, Preventive and Personalized Medicine, Diabetes mellitus, Risk, Guidelines, Patterns, Screening, Modeling, Patient Stratification

## Abstract

Missing values are common in health research and omitting participants with missing data often leads to loss of statistical power, biased estimates and, consequently, inaccurate inferences. We critically reviewed the challenges posed by missing data in medical research and approaches to address them. To achieve this more efficiently, these issues were analyzed and illustrated through a systematic review on the reporting of missing data and imputation methods (prediction of missing values through relationships within and between variables) undertaken in risk prediction studies of undiagnosed diabetes. Prevalent diabetes risk models were selected based on a recent comprehensive systematic review, supplemented by an updated search of English-language studies published between 1997 and 2014. Reporting of missing data has been limited in studies of prevalent diabetes prediction. Of the 48 articles identified, 62.5% (*n* = 30) did not report any information on missing data or handling techniques. In 21 (43.8%) studies, researchers opted out of imputation, completing case-wise deletion of participants missing any predictor values. Although imputation methods are encouraged to handle missing data and ensure the accuracy of inferences, this has seldom been the case in studies of diabetes risk prediction. Hence, we elaborated on the various types and patterns of missing data, the limitations of case-wise deletion and state-of the-art methods of imputations and their challenges. This review highlights the inexperience or disregard of investigators of the effect of missing data in risk prediction research. Formal guidelines may enhance the reporting and appropriate handling of missing data in scientific journals.

## Review

### Background

Missing values on participants’ characteristics are common in healthcare research and are often non-optimally handled and/or reported in prediction research. Inappropriate handling of missing data can lead to a poor model performance at the model development stage and mislabelling of the model at the external validation stage. It is therefore recommended that in predictive research, investigators strive to examine the patterns of missing values in their database to aid in classification of such information, use a valid approach to dealing with the missing data and include the description in their final report [1]. Predictive research is an area in which handling of missing data is of utmost importance. Indeed, simple risk prediction models based upon non-invasively measured predictors are increasingly advocated in population-based strategies for screening prevalent undiagnosed diabetes, particularly in low and middle income countries where undiagnosed diabetes is very common [2]. Accordingly, many prevalent diabetes risk prediction models have been developed over the last decade to convey this new thinking. Available models, however, remain specific to the population from which they were developed, until evidence of their good performance during external validations studies in different settings becomes available [3].

In this paper, we critically review the patterns of missing data and approaches to dealing with them, with a focus on predictive modeling. For illustrative purpose, we investigated how missing data have been reported and handled in predictive modeling, through a systematic review of studies on the development and/or validation of prevalent diabetes risk model. We hypothesized that the level of reporting and extent of imputation in studies of undiagnosed diabetes model development and validation would be poor.

### Methods

Building on a recent comprehensive review article on diabetes risk prediction models by Brown et al. 2012 [4], additional relevant articles were identified through a search of electronic database PubMed using the key terms ‘undiagnosed’, ‘diabetes’, ‘risk’ and ‘score’ and a manual search through reference lists of eligible studies. We selected studies aimed at developing or validating a risk prediction model. The outcome had to be prevalent undiagnosed diabetes in adults (aged >18 years). Models excluded were those of incident risk prediction or requiring blood tests (on the grounds that prevalent diabetes risk prediction aims at simple screening). The data extracted included country/setting (including its income classification), population/ethnicity, source of data and if from a questionnaire whether self-administered or not, sample size, age range of participants and the presence of a discussion and action (or lack thereof) on missing data.

We aimed at providing the reader with instances of missing data, their reporting and attempts to handle these, as well as the challenges posed by each method. In some instances, because of the paucity of reports on handling missing data in studies of diabetes risk prediction, we used examples from other fields for greater understanding and clarity on a topic that has not received much attention.

### Results

#### Overview of included studies

**Figure 1:**
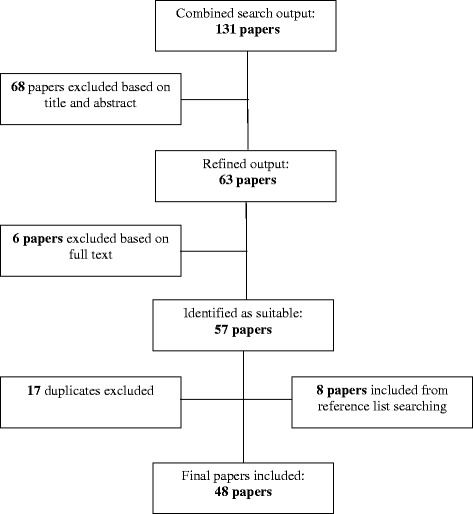
**Workflow summarizing the selection of papers.** Keywords: prevalent, diabetes, risk, prediction.

A total of 48 articles (26 were model development studies and 22 were external validations) were included (Figure 1). These are summarized in Table 1; published between 1997 and 2014 (mostly appearing in 2005–2010). The number and combination of predictors were variable, with age, sex, body mass index and waist circumference being the most commonly used variables. Models were developed and validated in 24 countries across 5 continents (none from Africa). Participants’ ethnicity was not always clearly stated, but number of studies included minority populations specific to locations (e.g. Asian and Black participants in a study conducted in the Netherlands) [5-10]. Administrative data was the most common source of data (30, 62.5%), from existent healthcare [11,12], governmental organization [9,13-15] or research settings [5,10,16-37]. The study sample sizes varied from 429 [28] to 68,476 [38]. Finally, the age of participants ranged from 18 to 94 years.

**Table 1:** **Characteristics of 48 included studies of undiagnosed diabetes risk prediction models**

**Author**	**Year**	**Validation or development**	**Location of study (income)**	**Ethnicity**	**Time of data collection**	**Type of data/self-administered**	**Size of study population**	**Age range**	**Missing data status**				
									**Reporting of missing data**	**Handling of missing data**			
									**None**	**Percent**	**None**	**Deletion**	**Imputation**
Adhikari et al. [39]	2010	Validate	India (L/M)	/	Current		551	>20	X		X		
Akyil et al. [40]	2014	Validate	Turkey (L/M)	/	Current		702	/	X		X		
Al Khalaf et al. [41]	2010	Develop	Kuwaiti (L/M)	Caucasian	Current	X	562	>20	X		X		
Al-Lawati et al. [16]	2007	Develop	Oman (H)	Caucasian	Existing		4,881	>20	X			X	
Baan et al. [17,42]	1999	Develop	Netherlands (H)	/	Existing	X	1,016	55–75	X		X		
Bang et al. [18]	2009	Develop	USA (H)	/	Existing		5,258	>20		X		X	X
Bergmann et al. [43]	2007	Validate	Germany (H)	/	Current		526	41–79	X		X		
Bindraban et al. [5]	2008	Develop	Netherlands (H)	Asian, Black, Caucasian	Existing		1,434	35–60		X		X	
Chaturvedi et al. [19,44]	2008	Develop	India (L/M)	/	Existing		4,044	35–64	X		X		
de Leon et al. [45]	2008	Develop	Canary Islands (H)	Caucasian	Current		6,237	18–75	X		X		
de Sousa et al. [13]	2009	Develop	Brazil (L/M)	Multi-ethnic	Existing	X	1,224	>35	X		X		
Franciosi et al. [20]	2005	Validate	Italy (H)	/	Existing	X	1,377	55–75		X		X	
Gao et al. [46]	2010	Validate	China (L/M)	Asian	Current		1,986	20–74		X		X	
Ginde et al. [6]	2007	Validate	USA (H)	Caucasian, African-American, Hispanic	Current		604	/	X			X	
Glumer et al. [21]	2004	Develop	Denmark (H)	/	Existing		6,784	30–60		X		X	
Glümer et al. [22]	2005	Validate	Australia/Denmark (H)	/	Existing		7,079/6,270	30–60		X		X	
Glumer et al. [23]	2006	Validate	Global	Multi-ethnic	Existing		29,758	/	X			X	
Gray et al. [24]	2010	Develop	UK (H)	Caucasian, Asian	Existing		6,186	40–75		X		X	
Gray et al. [25]	2013	Develop	Portugal (H)	/	Existing		3,435 (18–94)	18–94		X			X
Griffin et al. [11]	2000	Develop	UK (H)	Caucasian	Existing		1,077	40–64	X		X		
Hanif et al. [47]	2008	Develop	UK (H)	Asian	Current		435	20–75	X		X		
Heianza et al. [26]	2013	Develop	Japan (H)	Asian	Existing		7,477	18–88		X		X	
Heikes et al. [27]	2008	Develop	USA (H)	Representative of USA population	Existing		7,029	>20		X		X	
Heldgaard & Griffin [48]	2006	Develop	Denmark (H)	/	Current	X	1,355	20–69	X		X		
Keesukphan et al. [28]	2007	Develop	Thailand (L/M)	/	Existing		429	18–81	X		X		
Ko et al. [12]	2010	Develop	China (L/M)	Asian	Existing		7,695		X		X		
Ku & Kegels [49]	2013	Validate	Philippines (L/M)	/	Current		1,789		X		X		
Lee et al. [29]	2012	Develop	Korea (L/M)	/	Existing		9,602	>20		X		X	
Li et al. [50]	2009	Develop	Germany (H)	/	Current		921	14–93	X		X		
Lin et al. [51]	2009	Validate	Taiwan (H)	Asian	Current		2,759	>18	X		X		
Lindstrom et al. [14]	2003	Develop	Finland (H)	/	Existing	X	4,435	35–64	X			X	
Liu et al. [15]	2011	Develop	China (L/M)	/	Existing		1,851	40–90	X		X		
Mohan et al. [30]	2005	Validate	India (L/M)	Asian	Existing		2,350	>35	X		X		
Park et al. [31]	2002	Validate	UK (H)	Caucasian	Existing	X	6,567	39–78	X			X	
Rahman et al. [32]	2008	Validate	UK (H)	/	Existing		25,639	40–79		X		X	
Ramachandran et al. [33]	2005	Develop	India (L/M)	Asian	Existing		10,003	>20	X		X		
Rathmann et al. [34]	2005	Validate	Germany (H)	Caucasian	Existing		1,353	55–74	X		X		
Robinson et al. [7] Rolka et al. [8]	2011	Develop	Canada (H)	Caucasian, Aboriginal, Asian, Black, Hispanic	Current		6,475	40–74		X		X	X
Ruige et al. [35] Saaristo et al. [52]	2001	Validate	USA (H)	Hispanics, Caucasian, Black, Native American	Current		1,471	>20		X			X
Spijkerman et al. [9]	1997	Develop	Netherlands (H)	Caucasian	Existing	X	2,364	50–74		X	X		
Ta et al. [53]	2005	Validate	Finland (H)	/	Current supplemented with existing	X	2,966	45–74		X			X
Tankove et al. [54]	2004	Validate	UK (H)	Black, Asian	Existing		803	40–75	X			X	
Winkler et al. [38]	2010	Validate	Vietnam (L/M)	/	Current		721	30–70	X		X		
Witte et al. [36]	2011	Validate	Bulgaria (L/M)	/	Current		2,169		X		X		
Zhang et al. [10]	2012	Validate	Hungary (L/M)	/	Current		68,476	>18	X			X	
Zhou et al. [37]	2010	Validate	UK (H)	Caucasian	Existing		6,990	35–55		X		X	
Zhang et al. [10]	2014	Validate	USA (H)	Caucasian, Black	Existing	X	20,633	>20	X			X	
Zhou et al. [37]	2013	Develop	China (L/M)	/	Existing		41,809	20–74		X		X	

#### Source of missing data in predictive research

**Figure 2:**
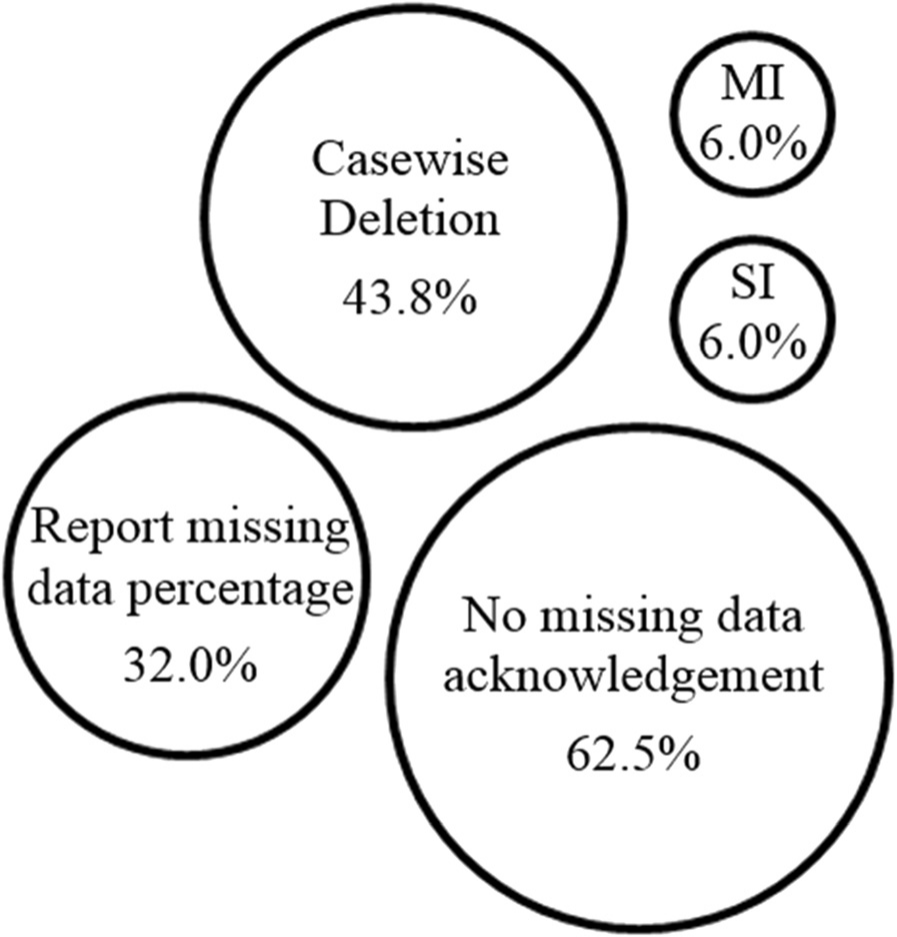
**Graphical representation of handling of missing data from the 48 selected studies.** *MI* multiple imputation, *SI* single imputation.

Figure 2 summarizes reporting and handling of missing data. The chief reasons for missing data are study design, participant characteristics, measurements characteristics, data collection and management and chance. These may occur alone or simultaneously within a study, with data missing for several different reasons acting additively.

#### Study design

The reviewed studies were cross-sectional. No study design can eliminate missing data, but the probability of missing data varies across designs, with longitudinal studies carrying a higher likelihood of missing data than cross-sectional studies. In longitudinal studies, a greater burden on the participants increases the likelihood of missing data, through the duration of the study, repeated measures, long questionnaires and painful procedures. With lengthy and cumbersome procedures, participants are prone to respond poorly or dropping out altogether. Indeed, Rolka et al. had high missing percentages for the invasive collection of a finger prick, fasting and 2-h post-load blood collection (0.2%, 26.0% and 27.0% of missing data, respectively), as the study design required three invasive and burdensome diagnosis tests.

#### Participant characteristics

Non-response to questions may be associated with personal characteristics of the participants, where the reason is an inaccuracy in information processing or refusal to provide information. Information processing may be related to the language and comprehension levels of the participant. Beliefs and the attitude towards the research topic or particular item collected are important in non-response due to refusal. All studies that reported some form of missing data values were conducted in high income countries; except three studies undertaken in China [37,46] and Korea [29], all published after 2010.

#### Measurement characteristics

The collection of quantifiable predictors can lead to missing data in a variety of ways. Observations may be lost due to malfunctioning equipment. The complexity, length and invasiveness of the measures may also lead to participants opting out of particular tests (e.g. oral glucose tolerance test). Finally, for predictors that are measured in a laboratory, errors in the pre-analytical sample collection and analytical testing can result in random missing data (e.g. incorrect blood collection tube selection or extended waiting time before analysing blood glucose sample, where glucose is lost through glycolysis). Demographic or behavioral information may be collected via questionnaires through an interview of or self-administration by the participant. Self-administration is limited by the lack of supervision thus the likelihood of respondent error, ultimately increasing missing data. Only three articles that included self-administrated questionnaires, also reported missing data [20,35,52]. Missing data was as high as 9% for body mass index and waist circumference in the study by Saaristo et al. [52] and 15.3% for the oral glucose tolerance test and 15.7% for questionnaire data in that of Franciosi et al. [20].

#### Data management

Poor management of data can result in the loss of data obtained from all participants. This may be due to the data transfer process from one format to another, such as the exclusion of individual values due to unclear writing, unconventional answers or inadvertently missing questionnaire items. Disorganized or poor data storage can also result in lost data through unsystematic filing and communication, or faulty or non-existent back up files. Of the 17 articles reporting missing data, 13 of these were studies using existing databases, all developed for research [5,18,20-22,24,26,27,29,32,35-37]. Although administrative data has its own issues, the reduced response burden, the possibility of a large sample size and comparatively low costs make this an increasing popular choice of data collection. *De novo* data collection requires the correct preparation, validation and processing of the survey to limit missing data. The two articles that reported missing data above 20% were based on new data collection [7,8].

#### Chance

Despite investigators’ best efforts to prevent missing data through a study design, data collection and measurements and subsequent management of the data, missing data can still occur by chance. This does not produce a bias; however, large amounts of data may be missing if multiple chance events occur which produces its own sets of problems such as reduction in statistical power [55].

#### Reporting of missing data

Missing data was frequently poorly handled with 62.5% of the articles not mentioning whether missing data was encountered and, if there was, how it was treated. Sixteen articles (33.3%) stated the missing data percentage, with two testing the effect on the final dataset but not reporting missing data details [24,27]. However, from the reporting, it is difficult to determine the type of missing data, as this was not investigated.

#### Types of missing data

Missing data can be classified as ‘missing completely at random (MCAR)’, ‘missing at random (MAR)’, and ‘missing not at random (MNAR)’, where the reason for missing data differs [56-60]. Identifying the nature and pattern of missing data allows the researcher to correctly choose a data imputation method, which is based on the assumptions about the patterns of missing data.

#### Missing completely at random

Data is MCAR where the random subset of observations missing will have similar distributions to observed values [56]. The reasons for missing are unrelated to characteristics or responses of the subjects. Missing completely at random is a strict assumption and can be tested for. Little et al. [61] provided a statistical test of the MCAR assumption, where a significant chi-square test indicates that the data are not MCAR. Examples of MCAR include administrative errors or laboratory accidents that occur at random.

#### Missing at random

Missing data is described as MAR when the missing data is conditional. The missing observations commonly depend on observed characteristics not missing, with systematic differences between the missing and observed data [1,62]. The assumption is fulfilled if the missing values are related only to measured, not unmeasured values. MAR examples include increased missing data in elderly individuals, subjects from a certain region or from a different calendar time. This is illustrated by Robinson et al. [7], where smoking status was only available for selected collection sites, as this question was added to the questionnaire during the last phase of data collection, resulting in a large percentage of item-missing data.

#### Missing not at random

Missing data that are not random are related to unobserved participant’s characteristics [56]. This type of missing data is problematic and imputation is not sufficient. An example of MNAR is the selective non-response by a subject, e.g. sexual orientation or weight where the association with social image may cause people to avoid or underestimate the answer.

#### Patterns of missing data

None of the selected articles on the prediction of prevalent diabetes risk discussed nor graphically presented patterns of missing data nor offered reasons for the missing data. In general, there are three patterns of missing data, namely univariate, monotone and arbitrary [63].

#### Handling of missing data

In existing studies of diabetes risk prediction, 21 (43.8%) stated all individual missing data were excluded from the study analysis, conducting complete case analysis. Two articles used simple imputation to overcome missing data [7,52] and two made use of multiple imputation [8,25], while a single article undertook both imputation methods [18]. Saaristo et al. stated the missing data percentage for the most commonly missing data (9% for both BMI and waist circumference), both of which were simply imputed with mean substitution [52]. Robinson et al. used a number of deletion and imputation methods [7]. Waist circumference (6% missing) was imputed with mean substitution, while family history (13%) was dealt with by the substitution of ‘no’ for unanswered questions. Case-wise deletion was undertaken for all other predictors of missing data, 3.9% of participants were excluded. Finally, smoking was excluded as a predictor all together due to the large percentage of missing data (35.0%).

Bang et al. used a complete case analysis for predictors with missing values as the missing data proportion was considered ‘small’, although not stated [18]. Multiple imputation was done for a family history of diabetes. Perhaps significantly, the studies with low missing data rates or few variables with missing data undertook multiple imputation as a solution. Rolka et al. reported a full dataset apart from only three predictors with missing data, namely postprandial time (3.0%), fasting blood glucose (26.0%) and oral glucose tolerance test (27.0%) [8]. Finally, Gray et al. described minimal missing data for the majority of predictors ranging from 0.1% for current hypertension to 1.7% for smoking status, apart from statin use (36%) [25]. The effect of missing data on both the modeling process and the final model chosen was assessed. Another article did not state the missing data proportion, but rather the overall effect of missing data, which was to underestimate the prevalence of prediabetes and undiagnosed diabetes by approximately 2% and 1.5%, respectively [27]. None of the three models using multiple imputation stated the details of the method [8,18,25], such as the number of imputations or the variables included in the imputation model. We herein discuss the key fundamental aspects of the various methods to dealing with missing data, which were seldom or inappropriately undertaken as mentioned above.

#### Proportion of missing data and impact on the method for handling missing data

A proportion (considered here as the proportion of subjects having *any* predictors missing) of ≤0.05 is considered the cut-off for no or simple imputation without sacrificing results [64]. A missing data proportion between 0.05 and 0.15 requires investigation of predictor relationships. If the predictor with missing values is unrelated to all of the other predictors, simple imputation is considered reasonable; else, conditional mean or stochastic regression is the minimum. Once missing data proportion is ≥0.15, multiple imputation becomes imperative.

#### Methods for dealing with missing data

##### Problems with simple alternatives to data imputation

Common in predictive modeling is the case-wise deletion of individuals with data missing for the required model predictors. Complete case analysis, or list-wise deletion, removes all subjects with missing values for any possible predictors to be used in risk models [65,66]. Alternatively, available case analysis, or pairwise deletion, includes subjects with complete data for the predictors to be included in the final model but who have missing data for other predictors not considered in the model [1]. List-wise or case-wise deletions lead to reductions in sample size, and as a consequence, a reduction in statistical power, increase in standard error, and bias and imprecision in the regression coefficient estimates is introduced if the data is not MCAR [67-69]. Furthermore, when more than one prevalent diabetes risk prediction models are to be validated in a new population, it is difficult to interpret the results when the number of subjects may vary across the analyses [1].

##### Imputation

Imputation of missing values is the process of replacing these values with accurate parameter estimates [70]. Imputation aims at predicting missing values by obtaining values through relationships within and between variables. In general, individuals should only be discarded if there is a missing predictor of overriding importance that cannot be reliably imputed from other information [1]. Table 2 details available imputation methods, namely single and multiple imputation, and their implementation in R statistical software. Single imputation (SI) includes simple imputation, conditional mean imputation, stochastic regression imputation and hotdecking, with each of these method having its own advantages and drawbacks.

**Table 2:** **Details of imputation options**

	**Theory**	**Package in R**
Single imputation methods		
Simple imputation	In a predictor (X) which is unrelated to all other X’s, substitution replaces all missing continuous values with the mean (or median) of all participants who have a valid value or the mode for categorical predictors [71].	Mean substitution is easily implemented with the package ‘*Hmisc*’ of R statistical software through the function ‘impute (x, fun = mean)’ where x is the predictor of interest [72].
	Simple imputation reduces variability and correlation estimates by ignoring relationships between variables but assumes MCAR. Regression coefficients are biased towards 0 (zero) since the outcome (Y) is not considered [1].	
Conditional mean imputation	Regression imputation assumes strong relationships between the X to be imputed and the independent X’s used in the univariable or multivariable regression formula [1,66,73]. An imputation model is made to predict the missing values when X is related to the other X’s, this method is far more efficient [74-76]. Conditional mean imputation leads to a weakening of the variance and overestimation of the model fit and correlation estimates. The outcome (Y) should not be included in the imputation model to prevent over exaggeration of the strength of relationship between X and Y [1].	Conditional mean imputation can be implemented in R through the creation of a regression model and the subsequent inbuilt ‘*predict*’ function.
Stochastic regression imputation	An alternative to conditional mean imputation, stochastic regression imputation includes a random element to the prediction of values, highlighting the uncertainty of imputed values [73]. A random draw is taken from the distribution of predicted values, which allows for the inclusion of the outcome in the prediction model.	This can be implemented with the ‘*mice*’ package for R via the command ‘*mice.impute.norm.nob*’ [77].
Hotdecking	Hotdecking replaces the missing value of an individual with a random value from a pool of individuals who are matched to the missing individual by predictors, the ‘deck’ [78,79]. These deck predictors may be researcher-determined or a correlation matrix may be used to determine which the most highly correlated predictors are. The standard error is better approximated through the hotdeck procedure than simple imputation.	The command ‘*hotdeck*’ of the R package ‘*VIM*’ can implement the hotdecking [80].
Multiple imputation methods		
Markov chain Monte Carlo (MCMC)	Multivariate normal imputation assumes a multivariate distribution and the MCMC algorithm is used to obtain imputed values and allow for uncertainty in the estimated model predictors [81]. MCMC describes a group of methods that use Markov chains to generate pseudorandom draws from probability distributions.	The command ‘*mcmcNorm*’ of the R package ‘*MCMCglmm*’ can implement MCMC approach to multiple imputation [82].
Maximum likelihood	The expectation-maximization (EM) algorithm, also called joint modeling, assumes a multivariate distribution. First a set of parameter values that produces the maximum likelihood are identified from the conditional distribution; values that would most likely have resulted in the observed data [77,83]. New parameter estimates are randomly drawn from a Bayesian posterior distribution, the distribution of unobserved values conditional on observed data [84]. Bootstrap procedures are employed to obtain standard error estimates, correcting for bias associated with non-normality.	The package ‘*Amelia’* in R implements bootstrapping algorithms to give EM results [85].

Multiple imputation (MI) describes the production of multiple complete datasets derived from the initial dataset with missing values [86]. Statistical models are used to fill the missing data a number (*m*) of times to generate *m* complete data sets. The multiple datasets add variability, increasing accuracy for both sampling and imputation, and the number of imputed datasets is usually set to 5 or 10 [87]. The datasets are analyzed separately using standard procedures, yielding multiple estimates which are then combined appropriately [88]. The first stage requires an imputation algorithm, while the combining of the analysis results of the multiple datasets requires an alternate pooling algorithm. Imputation algorithms may be univariate methods for monotone missing data such as predictive mean matching [89], propensity methods [90] or logistic regression; or for more complicated missing data, the multiple imputation by chained equations (MICE) or expectation-maximization (EM) algorithm have been proposed. Multiple imputation methods for non-monotone missing patterns using chained equations requires the decision of whether to use Markov chain Monte Carlo (MCMC) or fully conditional specification (FCS) methods. Expectation-maximization has yet to become that popular in medical applications but merits discussion and use.

Multiple imputation is time, labor and computationally intensive, and in case of small amount of missing data, researchers must decide on the use of this method or alternative methods [62,68]. The combination of lack of guidelines, imputer burden and perhaps lack of knowledge makes researchers hesitant to undertake MI. This hesitation is encouraged if MI is not going to be carried out successfully, with the failure to combine the final *m* datasets or leaving out of important predictors in the MI model.

##### Dealing with missing data in validation studies

The implementation of a model in an alternative population to that in which the model was developed requires prior validation. Differences between the development and validation datasets can be expected, with predictors possibly missing altogether, hindering validation of the model. This can be handled in a variety of ways, all which will have an effect on model performance or final model selection. Missing predictors can be dealt with by excluding models which contain any predictors not collected in the study. This limits the possibility of finding an existing model that may have suitable performance in the new population. Alternatively, the model may be selected for validation, but predictors in the model will be excluded from the model formula. This method could be improved by the substitution of a missing predictor with a reliable proxy variable, preventing model and predictor exclusion. Of the 22 validation studies, 11 (50%) used case-wise deletion of individuals or predictors in dealing with the missing data, with only a single article using mean imputation [52] and another multiple imputation [8].

### Discussion

Dealing with missing data is a complex undertaking, which is not yet common place in medical research. Indeed, for studies of development and validation of undiagnosed diabetes risk models, we found inconsistent reporting of missing data, with investigators frequently ignoring or failing to handle missing data appropriately. Despite the availability of a wide range of methods for handling missing data, only a handful of studies used the statistical modeling procedures. When imputation was undertaken, the reporting of the imputation procedures was often incomplete. Although multiple imputation is becoming more accessible in research, only three studies used this method with no details of the method being provided. Despite an increased interest in recent years in the need for understanding and appropriately handling missing data, the scarcity of information on these issues points to the widespread failure to understand the significance of the problem among medical researchers, hence the need to more formally address this issue.

In an effort to understand the lack of reporting and correct data handling in these studies, it must be noted that many imputation methods have mainly been developed theoretically and tested by statisticians. Medical professionals without any experience in statistics may struggle or chose not to undertake imputation procedures for missing data. Suggested reporting guidelines state the inclusion of the number of missing values, along with the reasons for the missing data, and the important differences between individuals with complete and incomplete data [91]. These guidelines can be useful for journal editors and authors alike, as hitherto the full impact of missing data on the research results is not usually considered.

Our review has limitations that merit consideration. Although we aimed to comprehensively review all papers on development and validation of undiagnosed diabetes risk prediction models, given that we relied on a single review article with a simple supplemental search, we may have missed some relevant studies. Furthermore, MI was not widely accessible prior to 1997 (the earliest date of publication of the included articles) so papers published immediately after this are more likely to have used complete case analysis or single imputation [92].

## Conclusions

This review highlights the inadequate reporting and handling of missing data in prevalent diabetes prediction research. Appropriate understanding, interpretation and efficient handling of missing data in medical research are essential, as incomplete data and the less than ideal methods in dealing with this can severely affect study estimates and other inferences in general. Publication of formal guidelines on the uniform reporting of missing data and methods for handling them at the analysis stage is warranted. These guidelines should be accessible to all levels of practitioners and researchers to allow for easy implementation, ultimately enhancing the validity of reported results in all spheres of prediction research.
